# Antenatal Care Utilization and Delivery Location: Evidence from Postpartum Women in Enugu, South East Nigeria

**DOI:** 10.21106/IJMA_381_2026

**Published:** 2026-08-04

**Authors:** Innocent Anayochukwu Ugwu, Calistus Obiora Nevo, Malachy Nwaeze Ezenwaeze

**Affiliations:** Department of Obstetrics and Gynaecology, College of Medicine, Enugu State University of Science and Technology (ESUT) and ESUT Teaching Hospital, Parklane, Enugu, Nigeria

**Keywords:** Antenatal Care, Birth place, Choice of Place, Skilled Birth Attendants, Traditional Birth Attendants, Women

## Abstract

**Background and Objective:**

Maternal mortality is high in sub-Saharan African countries. This death can be prevented by increasing the percentage of deliveries attended by skilled birth attendants (SBA). In Nigeria, most women still deliver with traditional birth attendants (TBA), leading to complications and death. This study examined factors associated with antenatal care (ANC) and delivery location among recently delivered women attending an immunization clinic in Enugu, Nigeria.

**Methods:**

This was a cross-sectional survey involving 501 participants. A pretested questionnaire was used to collect data through purposive sampling. Independent variables included the socio-demographic characteristics. The dependent variable was the choice of delivery location. Demographic characteristics and descriptive statistics were reported as percentages and frequencies. Associations between the variables were analyzed using chi-square testing. A two-sided p value below 0.05 was considered statistically significant.

**Results:**

Most participants attended ANC (92.8%) and delivered (90.6%) in a healthcare facility with SBA. The most common person influencing the choice of delivery location was “self” (54.8%). The most considered factor in choosing a place of delivery was the competence of the doctor/midwife (85.9%). Significant associations were found between choice of place of delivery and age (*ꭓ*^2^ = 11.403; p = 0.010), educational status (*ꭓ*^2^ = 61.012; p = 0.001), occupation (*ꭓ*^2^ = 14.931; p = 0.002), and monthly income (*ꭓ*^2^ = 44.440; p < 0.001).

**Conclusion and Global Health Implications:**

Most women received ANC and gave birth in a healthcare facility, though a few used home, spiritual houses, and TBAs. The choice of care location is associated with personal, socio-demographic, economic, and hospital-related factors. The findings suggest that perceived staff competence and service availability may influence women’s decisions about where to seek maternity care.

## INTRODUCTION

Maternal mortality remains concentrated in low- and middle-income countries, where in 2023, approximately 92% of the 260,000 maternal deaths occurred, with sub-Saharan Africa carrying the largest share of global maternal deaths.^[1]^ Recent international estimates place Nigeria among the countries with the greatest number of maternal deaths, accounting for 28.3% of all the world’s maternal deaths, and reporting a “maternal mortality ratio of 993 per 100,000 live births.”^[2]^ About one out of every 22 parturients in Nigeria is at risk of death due to pregnancy, delivery, or during the postnatal period.^[3]^ Poor medical infrastructure, pregnancy complications, weak referral systems, a dearth of healthcare workers, and poor health-seeking behavior are associated with increased maternal mortality rates in many countries, including Nigeria.^[4]^ There is a further shortage of health care workers in low- and middle-income nations due to the brain drain of health workers (especially in Nigeria) seeking greener pastures and access to standard, up-to-date equipment to improve their competencies.^[5]^ Evidence has shown that the utilization of the skilled birth attendant (SBA) and the patronization of the health facilities during childbearing can improve delivery outcomes and decrease maternal death and obstetric complications.^[6,7]^ In a study done in northern Nigeria, only about 33.1% of deliveries took place in hospital facilities and were supervised by an SBA (including nurses, midwives, and doctors).^[7]^ Serious obstetric complications may arise without warning, underscoring the importance of timely access to skilled personnel and emergency obstetric care.

The percentage of births overseen by trained health professionals, often cited as an effective indicator of progress in maternal and newborn health globally, has remained stagnant over the last 10 years.^[8]^ In Nigeria, many women continue to deliver their babies at home and in locations that are not healthcare facilities, often without the presence of qualified health personnel, even with the availability of free newborn and maternal health services at public healthcare facilities in certain states.^[9–11]^ Some women attend antenatal care (ANC) in formal health facilities but give birth elsewhere, leaving a gap between antenatal contact and access to skilled care during delivery.^[12]^

Clinicians at the study hospital have observed that some women seek care from both formal facilities and unskilled providers during the same pregnancy. This observation prompted the present study. Some of them who had ANC with SBA in the public health centers end up delivering their babies with unskilled birth attendants. They are only referred due to complications to the same facility with skilled personnel where they initially registered. Oftentimes, they present late with severe complications that would have been prevented. This study therefore examined factors associated with ANC and delivery location among postpartum women attending the immunization clinic at Enugu State University of Science and Technology (ESUT) Teaching Hospital.

## METHODS

### Study Setting/Design

We conducted a facility-based cross-sectional survey from April through October 2025. Participants were recruited from the immunization clinic of Enugu State University of Science and Technology Teaching Hospital, a tertiary public hospital in Enugu, southeastern Nigeria. It is a public hospital that offers free maternal and immunization services and therefore has a high influx of mothers who come to access immunization for their children, irrespective of where they delivered.

### Sampling Population

Eligible participants were women who had delivered within the previous 3 months and attended the clinic to immunize their infants. The 3-month interval was strictly applied at each recruitment date to reduce recall errors regarding ANC and delivery experiences.

### Sample Size/Sampling Approach

Cochrane’s formula for a single proportion, using a 95% confidence level, an expected proportion of 0.50, and a 5-percentage-point margin of error, gave a minimum sample of 384. After allowing 10% for nonresponse, the target sample was 422. Recruitment continued until 501 completed questionnaires were collected, as more eligible participants and resources became available. We also needed to account for attrition, handle incomplete data that may be removed during data cleaning, and achieve high statistical power. Data collection used a purposive sampling technique.

### Ethics

Ethical approval was obtained from the Ethical Review Committee of the Enugu State University of Science and Technology Teaching Hospital, Parklane, with ethical clearance certificate number ESUTHP/C-MAC/RA/034/Vol. 4/34. Participation was voluntary, and written informed consent was obtained. Participants were free to withdraw from the study at any time. Respect for persons, justice, and beneficence were ensured. Confidentiality of information was ensured, and data were securely stored.

### Inclusion/Exclusion Criteria

All consenting mothers who brought their children for immunization and delivered within 3 months of the data collection date were included. This restriction was intended to reduce recall bias. Those who delivered more than 3 months before the data collection date, as well as women who came to immunize another person’s child, were excluded.

### Data Collection and Management

Data were collected with a pretested structured questionnaire. The questionnaire contained a mix of open- and closed-ended questions, specifically designed to collect information related to the study objectives. These include questions on sociodemographic characteristics, average monthly income, and number of children. Others include questions about perceived factors influencing the location of ANC/delivery, and multiple answers were allowed. Responses to perceived factors that influenced ANC/delivery at the health facility were recorded as “Yes,” “No,” or “Not sure.” Pretesting of the questionnaire was conducted with 10% of the sample at the antenatal clinic. Corrections and modifications of the questionnaires were made before the study. Research assistants were employed and trained on data collection techniques. Before participation, participants were thoroughly informed about the study, and written informed consent was obtained. A total of 800 women were approached; 200 of them were excluded, and 99 declined to participate. Information on socio-demographic characteristics, places of ANC and delivery, and factors determining their choices was collected.

### Data Analysis

Completed questionnaires were checked, coded, and entered into IBM SPSS Statistics version 25, and analysis was done. Independent variables included the socio-demographic characteristics such as religion, age, level of education, place of residence, marital status, family monthly income, and number of children. The primary outcome was ANC/delivery in a health facility. A health facility is any place where health care services are offered by trained health care professionals at different cadres to promote, restore, improve, or maintain people’s health. Receiving ANC/delivery in a health care institution in this study includes care in any of the maternity centers, primary health centers, private/mission hospitals, and specialist/teaching hospitals. Missing responses were coded as “99,” indicating no response. Demographic characteristics and descriptive statistics were reported as percentages and frequencies (univariate analysis). Unadjusted associations were assessed using Pearson’s chi-square test or Fisher’s exact test, as appropriate. A two-sided p value below 0.05 was considered statistically significant.

## RESULT

A total of 501 women were enrolled. The number of data points contributing to each analysis varied and is shown in the tables. Valid numbers and percentages were reported.

### Socio-demographic Characteristics

Among the 473 women with recorded age, 201 (42.5%) were 35–44 years old. Among respondents with occupation data, traders were the largest group, 135 (30.3%). Among the 232 women who reported income, 129 (55.6%) reported a monthly household income above *₦*100,000. However, slightly over half (269, 53.3%) of the women were unable to respond to the question about their monthly household income.

### Antenatal Care and Place of Delivery

Among 479 women with ANC data, 472 (98.5%) reported receiving ANC and 7 (1.5%) reported receiving none. Among women with location data, 453/488 (92.8%) received ANC in a health facility, and 444/490 (90.6%) delivered in a health facility.

### Factors Influencing Institutional Delivery

Other factors considered by participants when choosing the health institution for ANC or delivery included: competence of doctor/midwife (371/432, 85.9%), 24-hour presence of a doctor (341/406, 84.0%), and presence of a specialist obstetrician (316/392, 80.6%). Multiple responses were allowed. Valid numbers and percentages were reported [Table 3].

**Table 1:** Sociodemographic characteristics of respondents.

Variables	Frequency (n)	Valid %	Total % (n/501)
Age (years)		(n/473)	
15–24	30	6.3	6.0
25–34	151	31.9	30.1
35–44	201	42.5	40.1
≥45	91	19.2	18.2
Total valid (N)	473	100	–
Missing	28	–	5.6
***Grand total***	***501***	–	***100***
**Occupation of respondents**		(n/446)	
Unemployed	26	5.8	5.1
Trader	135	30.3	27.0
Teacher	75	16.8	14.9
Civil servant	108	24.2	21.6
Professional	102	22.9	20.4
Total valid	446	100	
Missing	55	–	11.0
***Grand total***	***501***	–	***100***
**Marital status**		(n/496)	
Single	4	0.8	0.8
Married	489	98.6	97.6
Separated	3	0.6	0.6
Missing	5	1.0	1.0
Total valid	496	100	
***Grand total***	***501***	–	***100***
**Family type**		(n/463)	
Monogamous	425	91.8	84.8
Polygamous	38	8.2	7.6
Total valid	463	100	–
Missing	38	–	7.6
***Grand total***	***501***	–	***100***
**Religion**		(n/487)	
Christianity	395	81.1	78.8
Islam/others	92	18.9	18.4
Valid total	487	100	–
Missing	14	–	2.8
***Grand total***	***501***	–	***100***
**Tribe**		(n/487)	
Igbo	417	85.6	83.2
Others	70	14.4	14.0
Total valid	487	100	–
Missing	14	–	2.8
***Grand total***	***501***		***100***
**Educational status**		(n/475)	
None/primary	23	4.8	4.5
Secondary	83	17.5	16.6
Tertiary	369	77.7	73.7
Total valid	475	100	–
Missing	26	–	5.2
***Grand total***	***501***	–	***100***
**Residence**		(n/453)	
Urban	418	92.3	83.4
Rural	35	7.7	7.0
Total valid	453	100	–
Missing	48	–	9.6
***Grand total***	***501***	–	***100***
**Number of children**		(n/474)	
1	202	42.6	40.3
2	161	34.0	32.1
3	111	23.4	22.2
Total valid	474	100	–
Missing	27	–	5.4
***Grand total***	***501***	–	***100***
**Average monthly income**		(n/232)	
<50,000	41	17.7	8.2
50,000–100,000	62	26.7	12.4
>100,000	129	55.6	25.7
Total valid	232	100	–
Missing	269	–	53.3
***Grand total***	***501***	–	***100***

**Table 2:** ANC and place of delivery.

Variables	Frequency (n)	Valid % (n/N)	Cumulative (total) % (n/501)
Received ANC		(n/479)	
Yes	472	98.5	98.5
No	7	1.5	1.5
***Valid total (N)***	***479***	***100***	–
Missing	22		
Grand total	501	–	100
**Place where ANC was received in last pregnancy**		**(n/488)**	
Home/spiritual houses	35	7.2	6.9
Maternity/primary health center	37	7.6	7.4
Teaching/state specialist hospitals	349	71.5	69.7
General/private/mission hospital	67	13.7	13.4
***Valid total***	***488***	***100***	–
Missing	13	–	2.6
Grand total	501	–	100
^a^ ***ANC attendance in health facility***	***453***	***92.8***	***90.4***
**Place where last baby was delivered**		(n/490)	
Home/spiritual house/traditional birth attendant (TBA)	46	9.4	9.2
Maternity/primary health center	22	4.5	4.4
General/private/mission hospital	126	25.7	25.1
Teaching/state specialist hospital	296	60.4	59.1
***Total valid***	***490***	***100***	–
Missing	11	–	2.2
Grand total	501		100
^b^ ***Delivery in health facility***	***444***	***90.6***	***88.6***
**Person who influenced choice of place of last delivery**		(n/462)	
Self	253	54.8	50.5
Husband	128	27.7	25.5
Mother/mother-in-law	32	6.9	6.4
Spiritual leader	10	2.2	2.0
Friends	21	4.5	4.2
Others	18	3.9	3.6
***Valid total***	***462***	***100***	–
Missing	39	–	7.8
Grand total	501	–	100

^a^ANC attendance in health facility = Those that attended at maternity/primary health center + general/private/mission hospital + teaching/state specialist hospital. ^b^Delivery in health facility = Those who delivered at maternity/primary health center + general/private/mission hospital + teaching/state specialist hospital.

**Table 3:** Factors which influenced delivery at the health facility.

Factors	Yes N (%)	No N (%)	Not sure N (%)	Total N (%)
Affordable cost of care	243 (61.8)	136 (34.6)	14 (3.6)	393 (100)
Religious reason	68 (19.2)	271 (76.6)	15 (4.2)	354 (100)
Competence of doctor/midwife	371 (85.9)	51 (11.8)	10 (2.3)	432 (100)
Nearness to house	141 (37.7)	225 (60.2)	8 (2.1)	374 (100)
Friendliness of staff	228 (59.2)	133 (34.6)	24 (6.2)	385 (100)
Availability of pediatricians	273 (70.7)	92 (23.8)	21 (5.5)	386 (100)
Availability of ambulance	118 (33.4)	200 (56.7)	35 (9.9)	353 (100)
Fear of blood transfusion	64 (18.3)	259 (74.2)	26 (7.5)	349 (100)
Transportation difficulty	41 (11.8)	297 (85.6)	9 (2.6)	347 (100)
Privacy	153 (42.8)	192 (53.8)	12 (3.4)	357 (100)
Prophesies of care outcome	292 (76.3)	79 (20.6)	12 (3.1)	383 (100)
Availability of blood transfusion services	163 (46.3)	164 (46.6)	25 (7.1)	352 (100)
Presence of specialist obstetrician	316 (80.6)	63 (16.1)	13 (3.3)	392 (100)
24-hour presence of doctor	341 (84.0)	52 (12.8)	13 (3.2)	406 (100)
Availability of health education	301 (78.2)	70 (18.2)	14 (3.6)	385 (100)
Fear of cesarean section	83 (23.7)	244 (69.5)	24 (6.8)	351 (100)
Availability of facilities for operative delivery	251 (67.1)	107 (28.6)	16 (4.3)	374 (100)
Teamwork among doctors	296 (77.3)	65 (17.0)	22 (5.7)	383 (100)

### Associations Between Whether Health Facility Care Was Sought After and the Respondent’s Characteristics

Significant associations were observed for age (p = 0.010), education (p = 0.001), occupation (p = 0.002), and income (p < 0.001). Facility delivery was reported by 87/91 women aged 45 years or older and by 24/30 women aged 15–24 years. Women with higher education were more likely to seek care at health facilities. Similarly, professionals reported facility delivery more often than other occupational groups. Higher-income earners also sought care at health facilities more often than others [Table 4].

**Table 4:** Associations between place sought for care and respondents’ characteristics.

Variables	Health facility n (%)	Others n (%)	Total	Chi-square	p-value
Age					
15–24	24 (80.0)	6 (20.0)	30	11.403	0.010
25–34	140 (92.7)	11 (7.3)	151	–	–
35–44	192 (95.5)	9 (4.5)	201	–	–
45+	87 (95.6)	4 (4.4)	91	–	–
**Education**					
Primary or less	16 (69.6)	7 (30.4)	23	61,012	0.001
Secondary	66 (79.5)	17 (20.5)	83	–	–
Tertiary	362 (98.1)	7 (1.9)	369	–	–
**Tribe**					
Igbo	387 (92.8)	30 (7.2)	417	0.000	0.988
Others	65 (92.9)	5 (7.1)	70	–	–
**Number of children**					
1	192 (95.0)	10 (5.0)	202	2.926	0.232
2	151 (93.8)	10 (6.2)	161		–
3	100 (90.1)	11 (9.9)	111		–
**Residence**					
Urban	394 (94.3)	24 (5.7)	418	1.801	0.180
Rural	31 (88.6)	4 (11.4)	35	–	–
**Religion**					
Christianity	362 (91.6)	33 (8.4)	395	4.273	0.040
Others	90 (97.8)	2 (2.2)	92	–	–
**Occupation**					
Teachers	71 (94.7)	4 (5.3)	75	14.931	0.002
Traders	118 (87.4)	17 (12.6)	135	–	–
Civil servants	105 (97.2)	3 (2.8)	108	–	–
Professionals	100 (98.0)	2 (2.0)	102	–	–
**Monthly income**					
<50,000	29 (70.7)	12 (29.3)	41	44.440	<0.001^a^
50,000–100,000	59 (95.2)	3 (4.8)	62	–	–
>100,000	129 (100.0)	0 (0.0)	129	–	–
**Family type**					
Monogamous	396 (93.2%)	29 (6.8)	425	0.062	0.803
Polygamous	35 (92.1%)	3 (7.9)	38		–

^a^Fisher’s Exact test reported.

## DISCUSSION

We examined factors associated with ANC and delivery location among recently delivered women attending an immunization clinic at a tertiary hospital in southeastern Nigeria.

### Antenatal Care and Place of Delivery

Notably, the majority of the patients (98.5%) received ANC. The high ANC attendance was similar to that reported in another hospital-based study.^[13]^ The similarity could be attributed to both studies being hospital-based and conducted in an urban center. Because both samples were drawn from health-service users, neither should be interpreted as population coverage. This finding was in contrast to the national estimate from the National Demographic and Health Survey, which reported that only 63% of women received ANC.^[14]^ The difference in findings may be because this study was hospital-based and conducted in an urban area, where attendees are more likely to be more educated and socioeconomically more advantaged than the national demographic survey, which included women in both rural and urban areas. However, up to 9.4% of participants did not attend ANC or attended places where they did not have the opportunity to access the services of a midwife, doctor, gynecologist, or any other person trained to provide ANC services. ANC offers a safety net for pregnant women. It assists in early identification and management of potential complications; monitoring of blood sugar, blood pressure, and other vital signs; educating women on breastfeeding, healthy habits, and newborn care; and provision of supplements such as folic acid and iron. When women do not attend ANC or attend in a place where there is no qualified health personnel, potential pregnancy complications often become undetected and untreated, resulting in severe consequences both to the mother and the newborn baby.

Although the majority of patients (90.6%) delivered in a health care facility, about 9.4% delivered at home, in spiritual houses, or with traditional birth attendants (TBAs), where qualified health personnel are not present. This finding is similar to that done in Nigeria, where it was shown that the majority (75%) of antenatal attendees delivered in a health facility, while 15% delivered with TBAs with insufficient skills to manage complications that may arise during delivery.^[15,16]^ The similarities in findings may be attributed to the fact that both studies were hospital-based and in urban areas. The high level of facility delivery may partly reflect the selected sample: all participants were attending an immunization clinic at a tertiary hospital, and most had also used formal ANC. ANC attendance has been associated with delivery in a health care facility.^[16]^ Women have greater opportunities to reinforce the health messages they receive with more ANC attendances, mainly because this higher number leads to better understanding and compliance.^[15]^ However, in studies comparing rural and urban utilization of skilled versus unskilled birth attendance, as in our study, urban dwellers use skilled birth attendance more than rural dwellers.^[17]^

### Factors Influencing Place of Delivery

This study showed that, to some extent, women and their husbands are responsible for the choice of delivery location. This is consistent with another study in southwest Nigeria, which also noted that spouses primarily determine the place of childbirth.^[13]^ Both studies were hospital-based, and participants were of similar cultural and socioeconomic backgrounds. Where couples choose to have their babies is not necessarily at random. Reasons for choosing a place may include quality of care and safety, cost and financial factors, access and convenience, social and cultural factors, trust and familiarity, as well as medical and obstetrics factors. In this study, the factors rated higher included the competence of obstetricians and midwives (85.9%), the presence of a specialist obstetrician (80.6%), the 24-hour presence of doctors (84.0%), and the availability of health education (78.2%).

In the unadjusted analysis, women’s delivery location in Enugu varied by age, education, occupation, and income, consistent with findings from other similar studies, which showed that certain socio-demographic factors influence women’s choice of place of delivery.^[18]^ Significant relationships between seeking institutional delivery and respondents’ characteristics were found for age (p = 0.010), education (p = 0.001), occupation (p = 0.002), and income (p = 0.001). Patronage of health institutions increased with age: 95.6% of those aged 45 and above preferred health institutions, compared with 80% of those aged 15–24. This finding is in agreement with the findings of other investigators in a similar setting, which also showed that older pregnant women preferred institutional/hospital delivery compared to younger ones in their study.^[13]^ Another study showed that older women aged 40–49 years were positively associated with hospital/institutional delivery, even after adjusting for other confounding variables.^[19–21]^ One possible explanation is that older women or their clinicians perceive greater obstetric risks such as antepartum hemorrhage, preeclampsia/eclampsia, preterm delivery, low birth weight, as well as diabetes in pregnancies. The awareness of this risk may drive older women to the hospital. Those with higher education and income, as well as professionals, had higher proportions seeking care at health institutions than those with lower education and income. This is also consistent with findings from investigators who demonstrated that women with higher education and higher income were significantly more likely to deliver in a health facility.^[22,23]^ These factors are all interrelated. Education improves women’s health literacy and risk perceptions, enables them to navigate the health system, and gives them greater decision-making power and autonomy. Education also correlates with professionalism, employment, and higher family income. In this study, conducted in an urban area, the majority of the women were professionally employed, had consistent incomes, autonomously chose their ANC and delivery sites, independently accessed health institutions for care, and received reliable monthly pay. These employed women, possessing a consistent income, were more inclined to autonomously decide their preferred locations for childbirth and to independently access health facilities for care.

### Limitations of the Study

There is a possibility of recall bias, as the women may not have been able to recall events that occurred several months before the survey. Restricting eligibility to women who had delivered within the previous 3 months may have reduced recall error. However, self-reported care location and decision-making remain subject to recall and social desirability biases. This study is limited by its cross-sectional design, which precludes causal inference. It cannot establish that the listed factors caused or influenced the choice of use of maternity services. The sample was drawn from an urban tertiary hospital immunization clinic. It may not represent women who use other facilities, deliver at home, do not attend infant immunization, or experience stillbirth or early neonatal loss. The findings should therefore not be generalized to Enugu State or Nigeria. A larger multicenter study with a higher sample size is needed.

## CONCLUSION AND GLOBAL HEALTH IMPLICATIONS

The majority of the women had ANC and delivered in health institutions, though a few used home, spiritual houses, or TBAs. The findings support continued attention to the perceived quality and availability of maternity services. Promoting institutional ANC/delivery through improvement in the perceived factors that attracted women to the various health facilities where they delivered will assist in reducing maternal death.

### Key Messages

1. Antenatal care and delivery rate in healthcare facilities is relatively high in Enugu compared to the general population in Nigeria.
2. Education, maternal age, and family income as well as quality and perceived quality of care are critical in healthcare facility delivery.
3. Community education and women’s empowerment, alongside improvement in the quality of care in healthcare facilities, will further enhance the use of skilled care during pregnancy and delivery.

**Acknowledgments:** The authors wish to express their appreciation to the nurses, resident doctors, and management staff of ESUT Teaching Hospital for their assistance during this study.

## COMPLIANCE WITH ETHICAL STANDARDS

### Conflicts of Interest

The authors declare no competing interests.

### Financial Disclosure

Nothing to declare.

### Ethics Approval

Ethical approval was obtained from the Ethical Review Committee of the Enugu State University of Science and Technology Teaching Hospital, Parklane, with ethical clearance certificate number ESUTHP/C-MAC/RA/034/Vol.4/34.

### Declaration of Patient Consent

Participation was voluntary, and written informed consent was obtained. Participants were free to withdraw from the study at any time.

### Use of Artificial Intelligence (AI) - Assisted Technology for Manuscript Preparation

The authors confirm that there was no use of Artificial Intelligence (AI) - Assisted Technology for assisting in the writing or editing of the manuscript, and no images were manipulated using AI.

### Disclaimer

None.
